# ESSENTIAL CAPABILITIES FOR MANAGING THE LONG-TERM CARE WORKFORCE: EVIDENCE FROM AN INTERNATIONAL SCOPING REVIEW

**DOI:** 10.1093/geroni/igad104.1069

**Published:** 2023-12-21

**Authors:** Philip Taylor

**Affiliations:** Federation University Australia, Ballarat, Victoria, Australia

## Abstract

Amid ongoing concerns about meeting the staffing needs of long-term care providers, arguably exacerbated by the COVID-19 pandemic, there is a need to better understand the skills and capabilities required of managers in the sector. Internationally, the management of the long-term care workforce has been somewhat neglected by both the research literature and recent enquiries into the sector. There is fundamental agreement that well-managed staff provide better care. The sector’s leadership and its workforce have been under scrutiny for some time and the context of COVID-19 has shone further light on their limited ability to respond to crises. It is recognised that operational deficiencies in terms of the organization and characteristics of the sector’s workforce as well as its socio-cultural positioning have contributed to sub-optimal functioning. This project collated evidence and recommendations that could inform policymaking among long-term care providers as well as policy and decision makers. An evidence review of relevant international gray and academic literature was undertaken and examples of successful models of management practice across a range of dimensions were identified and written up as vignettes. The paper reports key information regarding the building of capabilities in long-term care workforce management, including: current and future workforce considerations, value proposition, onboarding of staff, career development and retention. Recommendations regarding essential management capabilities in the long-term care workforce are set out with the intention of providing a basis for developing innovative and novel methods for improving and monitoring the quality and impact of management on the workforce.

